# Young children’s overestimation of performance: A cross‐cultural comparison

**DOI:** 10.1111/cdev.13709

**Published:** 2021-11-06

**Authors:** Mengtian Xia, Astrid M. G. Poorthuis, Qiang Zhou, Sander Thomaes

**Affiliations:** ^1^ Department of Psychology Utrecht University Utrecht The Netherlands; ^2^ Department of Psychology Wenzhou Medical University Wenzhou China

## Abstract

Western literature suggests that young children overestimate their performance across a range of tasks. Research in non‐Western cultures, however, is lacking. In 2019, 101 Chinese (52% girls) and 98 Dutch (49% girls) children, ages 4 and 5, were asked to estimate how well they would perform on both a motor and a memory task. Children from both countries overestimated their performance to the same extent ($\eta_{p}^{2}$ = .077 and .027 for the motor and memory tasks, respectively). They generally persevered in doing so despite receiving realistic performance feedback. Yet, children overestimated their peers’ performance about as much as their own performance, in some cases even more. This is the first demonstration of performance overestimation in children growing up in a non‐Western culture.

**Abbreviation**
ANCOVA: analysis of covariance

A Chinese proverb says that newborn calves are not afraid of tigers. Similarly, young children often seem undeterred by unfamiliar tasks and challenges. Research has shown that, in early childhood, children often feel overconfident about managing new tasks and challenges, and overestimate their competencies and performance (Lipko et al., 2009; Plumert, 1995; Shin et al., 2007; Yussen & Levy, 1975). However, this research has been conducted nearly exclusively in samples of children growing up in Western cultures, a limitation that applies to much of developmental science (Nielsen et al., 2017). Thus, the cultural generalizability of these results is yet unknown. This is important, especially in light of differences in the cultural values of self‐enhancement and modesty in Western and East Asian cultures. Here, we ask to what extent young children's self‐overestimation and its underlying psychological mechanisms generalize to children growing up in China. We investigate this question using a series of structured observations in young children growing up in mainland China and, as a comparison, their counterparts growing up in the Netherlands.

## Self‐enhancement and modesty across cultures

In general, culture is an important source of psychological and behavioral variation, and in particular in terms of self‐development (Henrich et al., 2010; Kline et al., 2018; Markus & Kitayama, 1991; Triandis, 1989; Q. Wang, 2006). Culture prescribes what is a “good person,” and cultural members, including children, try to live up to that ideal (Bornstein & Cheah, 2006; Gaertner et al., 2008; Triandis, 1989). In Western cultures (e.g., the United States, Northern Europe), social norms emphasize the importance of positive distinctiveness and personal success (e.g., Sedikides et al., 2015). Children are exposed, from a young age, to messages that convey it is ideal for them to be unique and stand out from others (Gürel & Brummelman, 2020; Thomaes et al., 2017; Young‐Eisendrath, 2008). For example, such messages are communicated through mass media emphasizing the importance of being “special,” adults encouraging social comparison and competition (e.g., in sports), and educational practices in schools such as singling out good performance (Gürel et al., 2020). Practices such as these both reflect and feed culturally shared ideals of attaining independence and agency in Western cultures.

In East Asian cultures (e.g., China, Japan), social norms more often emphasize the importance of interpersonal cohesion and harmony, of “fitting in” rather than “standing out” (Markus & Kitayama, 1991). Indeed, reflecting the Confucian proverb “haughtiness invites loss while modesty brings benefits,” modesty is a prevailing social norm in these cultures. As a disposition, modesty reflects a tendency for individuals to downplay their abilities or achievements, or at least refrain from self‐aggrandizement in order to maintain or promote social bonds (Kim et al., 2010; O’Mara et al., 2012). East Asian children are often familiarized with modesty as a social norm from an early age. For example, from early childhood, they learn not to present themselves to others in overly flattering ways, and to exercise restraint in communicating their accomplishments or good performance to others (Luo et al., 2013; Y. Wang & Ollendick, 2001; Wu et al., 2002; Xu et al., 2005). These socialization practices reinforce culturally shared ideals of personal integration and social connection.

From middle childhood, children learn to reason about modesty as a self‐presentational tactic that can benefit others’ evaluations of the self (Watling & Banerjee, 2007; Yoshida et al., 1982). Cultural differences in such reasoning emerge from this age. For example, East Asian children ages 7–11 rate the modest self‐presentations of their peers (portrayed in hypothetical scenarios) more favorably than their Western counterparts do (Heyman et al., 2011; Lee et al., 1997). Cultural differences also manifest in terms of actual modest behaviors. For example, in a modesty dilemma paradigm that provided children an opportunity to talk about a good deed they had done, East Asian children ages 7–11 were more likely to show modest behavior (i.e., falsely denying that they had done a good deed) as compared to their Canadian counterparts (Fu et al., 2016).

In rudimentary form, modest behavior may first appear at an even younger age. Already during the preschool years, children anticipate that they are being evaluated by others and they engage in various behavioral strategies to promote their reputational interests (Botto & Rochat, 2019; Heyman et al., 2021; Tomasello & Vaish, 2013). It is possible that the self‐presentations of East Asian preschoolers are shaped by prevailing social norms and socialization practices that emphasize modesty (Luo et al., 2013; Wu et al., 2002). Indeed, one study found that from around age 4, Chinese children already describe themselves in a more neutral or modest way than Western children do, who provide more favorable self‐descriptions (Q. Wang, 2004). To be sure, this finding does not mean that Chinese children necessarily hold less positive self‐concepts than Western children—rather, modest self‐presentations can be tactical. Although not yet tested in children, social psychological research has identified the tendency for Chinese adults to deemphasize the positivity of the self in their self‐presentations, even if they do positively evaluate themselves in response to indirect or allegedly private measures of self‐evaluation (Cai et al., 2011; Kim et al., 2010).

## Self‐estimation of competence and task performance in early childhood

Early work on young children's self‐estimation of competence and task performance focused on cognitive tasks. For example, Flavell et al. (1970) investigated how children in preschool and kindergarten (as compared to older children) estimate their performance on a memory task. They found that children in both of the youngest age groups overestimated their memory span (as compared to their actual memory span) prior to the task, more so than older children did. In fact, the overestimation effect in preschoolers and kindergarteners was 206% and 221%, respectively—they thought they would do more than twice as well as they actually did. Other work similarly found that preschoolers and kindergarteners overestimate their cognitive competencies, such as in terms of their performance on school tasks, and their understanding of mechanical devices and procedures (Mills & Keil, 2004; Stipek, 1981).

Similar research has examined young children's self‐estimation of competence and performance on motor tasks. Such self‐estimation is often dependent on children's perception of affordances (Gibson, 2014)—that is, they determine which actions are possible given their physical capabilities (e.g., body size or strength) and situational demands. The research found that young children (i.e., at least up until age 5 or 6) routinely overestimate what they are physically capable of. For example, they misjudge whether they are able to stand on steep slopes (Klevberg & Anderson, 2002), whether their hands fit through small openings (Ishak et al., 2014), whether their bodies fit through small doorways (Franchak, 2019), and whether they are capable of challenging motor tasks (e.g., removing a toy from a shelf standing on tiptoes; Plumert, 1995). Similarly, they predict they will achieve better on various motor tasks (i.e., jumping as far as possible, throwing a ball with accuracy) than they actually do (Schneider, 1998). In this latter study, the overestimation effect in preschoolers and kindergarteners was 140% and 118%, respectively, for the jumping task; and 161% and 162%, respectively, for the ball throwing task.

## What may account for self‐overestimation in early childhood?

Two main, though not mutually exclusive explanations have been offered to account for the apparent pervasiveness of young children's self‐overestimation. One is the “monitoring deficiency” account. According to this explanation, young children are not yet capable of reliably monitoring and retaining information on their abilities and their past performances, which means they do not have the cognitive means to accurately estimate their future performance (reviewed in Bjorklund & Green, 1992; Schneider, 1985). Another is the “wishful thinking” account. This explanation proposes that young children often fail to reliably distinguish between their wishes and expectations (Stipek et al., 1984). This would lead them to make performance predictions based on how well they would *want* to perform, rather than on how well they are actually able to perform, resulting in self‐overestimation (Lipko‐Speed, 2013; Schneider, 1998; Stipek et al., 1984).

Research in samples of Western children has challenged the monitoring deficiency account. For example, studies that assessed children's performance postdiction (i.e., performance recollection shortly after completing a task) found that even 4‐year‐olds are usually able to remember their performance on a previous task, but still, they remain overly confident when predicting their performance on a future task (Lipko et al., 2009; Schneider, 1998). This work thus suggests, different from what the monitoring deficiency account posits, that even young children typically can accurately monitor their task performance. However, they often fail to integrate this information into their estimates of their future performance. As some preschoolers stated when researchers showed them their past failures on a memory task: “If you give me a different list [of items to recall] like that, I could do it.” (Yussen & Levy, 1975, p. 507).

Other research in Western children did provide partial support for the wishful thinking account. For example, a number of studies found that preschoolers’ estimates of the performance of their peers are sometimes more accurate (i.e., less inflated) than their estimates of their own performance (Lipko et al., 2009; Schneider, 1998; Stipek et al., 1984). Moreover, when promised a reward for the good performance of their peers (i.e., so that good peer performance becomes desirable), preschoolers raise their estimates of their peers’ performance accordingly (Stipek et al., 1984). Thus, young children often make overly optimistic performance estimates when good performance is desirable. That said, research has also found that wishful thinking is context‐dependent and can account for overconfidence on some tasks, but not on others (Lipko et al., 2009; Schneider, 1998).

## The present study

Research shows that young children often overestimate their competence and task performance, but evidence has been obtained virtually exclusively in Western samples. We ask to what extent self‐overestimation can also be found in children growing up in an East Asian cultural context that highly values modesty. In a first cross‐cultural study of its kind, we examine young children's performance estimates in samples of Chinese and Dutch children, using both a cognitive and a motor task. We do so by tracking participants’ estimated and actual performance across task trials.

We also explore the psychological underpinnings of children's performance estimates, informed by the monitoring deficiency and wishful thinking accounts. We use multiple‐trial tasks and make salient how children perform, to be able to test the possibility that children's performance estimates gradually become more realistic as they gain experience and receive performance feedback. We ask children to estimate both their own and an unknown peer's performance, to test whether their judgments are more realistic when they have no investment in good performance.

We test children ages 4 and 5—an age at which (Western) children typically overestimate their competence and performance. We use behavioral assessments, rather than questionnaires or interviews, to assess children's performance estimation. This allows for direct cultural comparison and minimizes potential language confounds. We calculate self‐overestimation as the discrepancy between children's estimates of their performance just prior to the task, and their actual performance on the task.

We test the hypotheses that children (1) overestimate their performance on both tasks; (2) persist in overestimating their task performance across trials; and (3) overestimate their own performance more than they overestimate the performance of their peers. For each of the hypotheses, we explore potential differences between Chinese and Dutch children—our primary interest was in the overestimation of Chinese children, and we included a sample of Western children to allow direct cultural comparison.

We preregistered our hypotheses, design, targeted sample size, and analysis plan at aspredicted.org, [As Predicted: “A Study on the Phenomenon of Children's Overestimation” (#29787)]. In Supporting Information, we specify where and why we deviated from the preregistered analysis plan. We deviated from the preregistered analysis plan to reduce the risk of Type 1 error due to multiple testing. We conducted additional analyses to provide further evidence relevant to the hypotheses and we omitted one analysis that turned out to be superfluous in light of the research findings.

## METHOD

### Participants

We tested 101 children from China (52% girls) and 98 children from the Netherlands (49% girls). Participants were ages 4 and 5. We recruited participants for both samples, in the same way, using convenience sampling. We contacted (pre)schools to ask if they were interested in taking part in the study. If they were, we shared informed consent forms among parents of all students ages 4 or 5. We tested all children for whom we received consent. We conducted the study in the fall of 2019 (in both countries). The study was approved by the ethics board of the Faculty of Social Sciences, Utrecht University.

Chinese children's mean age was 4 years and 9 months (*SD* = 5.0 months, range = 50–71 months). They were recruited from a preschool in Wenzhou City, Zhejiang Province. The informed parental consent rate was 72%. Participants lived in an urban area. The school serves ethnically homogeneous, predominantly middle to upper class communities (in terms of family income and education level). Preschool education in mainland China aims to help children adapt to the school system and is mainly organized around structured and collaborative play.

Dutch children's mean age was 5 years and 0 months (*SD* = 6.1 months, range = 50–71 months). They were recruited from six primary schools across the Netherlands (in the Netherlands, most children start primary school at the age of 4). The informed parental consent rate was 63%. Participants predominantly lived in urban or suburban areas. The schools mainly serve ethnically homogeneous, middle‐class communities. Similar to preschool education in China, education in the first two grade years aims to help children adapt to school and mainly involves structured and collaborative play.

#### Data exclusion

We excluded the data of five participants (*n* = 1 and *n* = 4 Chinese and Dutch children, respectively) on the motor task, and the data of eight participants (*n* = 1 and *n* = 7 Chinese and Dutch children, respectively) on the memory task, from the pertaining analyses. Following our preregistered protocol, we excluded data either because they were incomplete (*n* = 1 and *n* = 7 for the motor and memory task, respectively), or because they deviated more than 3 *SD*s from the mean (*n* = 4 and *n* = 1 for the motor and memory task, respectively). Thus, we analyzed motor task data of *n* = 100 Chinese children and *n* = 94 Dutch children; and memory task data of *n* = 100 Chinese children and *n* = 91 Dutch children.

### Procedure

All participants performed the motor task first. To retain statistical power, we did not counterbalance the order of tasks. To limit possible carryover effects or fatigue, participants performed the memory task on another day (2–14 days later). The experimenters spoke participants’ native language (i.e., Mandarin or Dutch). All task instructions and responses to potential questions were standardized, translated, and back‐translated from English by bilingual speakers.

#### Motor task

**FIGURE 1:**
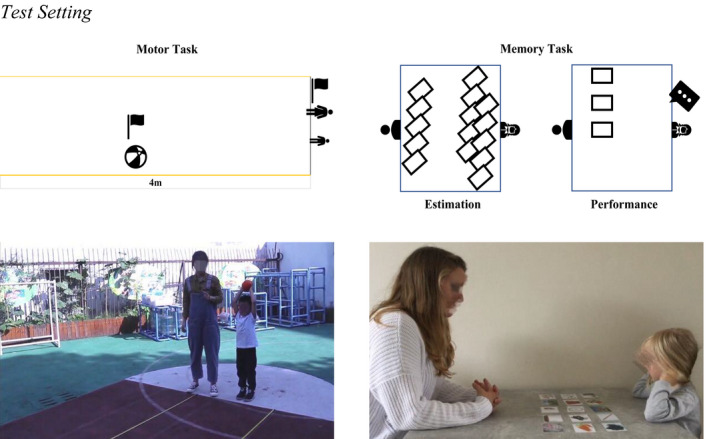
Test setting

We designed the motor task for the present study purposes. We aimed to design a task that was easy to understand for children this age. We kept task difficulty constant across trials (so that performance feedback on one trial can potentially inform children's subsequent performance estimate). We tested children individually in a spacious place on school grounds (Figure 1). We instructed them to stand in front of the starting line of a throwing field, which consisted of two parallel extended 4 m rulers, placed one meter apart, to mark the boundaries of the field. The experimenter handed the ball (i.e., 11 cm in diameter and 1 kg in weight) to the child and said, “Here you go. You can briefly hold the ball so you know a bit how it feels.” Then the experimenter took the ball back and asked: “How far do you think you can throw the ball? Could you put the flag somewhere on the throwing field to tell me?” After the child placed the green flag, we registered the distance from the starting line to the flag (i.e., Motor Self Estimate 1) and immediately removed the flag. We then asked the children to return to the starting line and lift the ball over their heads. The experimenter instructed the children: “When I count to three, you will throw the ball as far as possible, okay? Now, one, two, three.” The second experimenter observed where the ball first landed and recorded its distance from the starting line (i.e., Motor Self Performance 1), and placed a blue flag on the spot to provide children with feedback on their performance.

Next, with the blue flag still present, we asked children to place the green flag in the throwing field again, to indicate how far they thought they would throw the ball (i.e., Motor Self Estimate 2). The experimenters then removed both flags, asked children to throw the ball as far as they could, and placed the blue flag where the ball landed (i.e., Motor Self Performance 2). We repeated this procedure until participants had made four estimates, and we had recorded three ball‐throwing distances. Note that we included an estimate after the last ball throw to be able to test, for each ball throw, whether children learn from their previous performance and adjust their estimates.

Immediately after participants completed the above task, we showed them a video in which a child of about the same age, sex, and nationality performed the same task. We showed the video on a tablet computer, at the testing site. We introduced the peer with a common Chinese or Dutch name (i.e., Xiaoming or Xiaohong; Daan or Lisa) to be easily referred to. The experimenter paused the video just before the child in the video was about to throw the ball, and asked participants: “How far do you think […] can throw the ball? As before, can you put a green flag on the throwing field to tell me what you think?” After participants placed the flag, the second experimenter recorded its distance from the starting line (i.e., Motor Peer Estimate 1) and immediately removed it. Experimenters assisted participants to watch the video of the peer's performance and placed a blue flag to provide participants with feedback on the peer's performance (i.e., Motor Peer Performance 1).

We placed the blue flag where the ball landed in the corresponding trial when participants themselves took part in the ball throwing task (i.e., Motor Peer Performance 1 = Motor Self Performance 1; Motor Peer Performance *n* = Motor Self Performance *n*). This allowed us to directly compare children's performance estimates for themselves with those for their peers, unconfounded by any differences in actual performance. With the blue flag still present, we asked participants to place the green flag again to indicate how far they thought the child in the video would throw the ball the second time (i.e., Motor Peer Estimate 2). Then, the experimenters removed the two flags, assisted participants to watch the video of the peer's performance, and placed the blue flag (again, matching participants’ own previous performance on the corresponding trial). We repeated this procedure until participants had made four estimates and had watched the peer in the video throw the ball three times.

#### Memory task

We modeled the memory task after similar methodologies used in previous studies (Lipko et al., 2009; Lipko‐Speed, 2013; Shin et al., 2007). Again, we made sure that the task was fairly easy to understand for children this age, and we kept task difficulty constant across trials. We tested participants individually in a quiet and private room (Figure 1). We laid out a set of 15 blank cards on the table (previous work used 10–15 cards; we used 15 cards to ensure ample scope for children to overestimate their performance). The experimenter sat face to face with the child and said: “Next you will try to remember the same number of cards. But those cards will have pictures on them. How many cards do you think you can remember? Just leave the number of cards that you think you can remember on the table. You can give the rest of the cards back to me.” The experimenter recorded how many cards children left on the table (i.e., Memory Self Estimate 1) and then removed all cards.

Next, the experimenter showed the first of three sets of 15 picture cards. Each set contained 15 picture cards, and each picture corresponded to one of 15 themes (e.g., fruits, animals, musical instruments, toys). The experimenter laid out the picture cards on the table, one by one, and asked: “Can you tell me what it is when I show you the card?” Children were almost always able to name the pictures. If not, the experimenter informed them how to name the picture. We always followed children's own use of words—thus, if they used an incorrect word to name a picture, the experimenter did not correct them. Next, participants studied the picture cards, until the experimenter removed them after 15 s, and said: “Now you can tell me the name of each picture that you remember.” Each time the child recalled a picture correctly, the experimenter placed a picture card face down on the table. The experimenter encouraged children by saying “try again” or “think about it” when participants remained silent or seemed distracted for more than 5 s. When children said that they could not recall any more pictures or remained silent or distracted for more than 20 s, the experimenter ended the trial and said: “Okay! These are the card(s) that you remembered correctly.” The experimenter recorded the number of correctly recalled picture cards (i.e., Memory Self Performance 1).

Next, with the correctly recalled face‐down picture card(s) still on the table, we laid out another row that consisted of 15 blank cards, to allow children to estimate their performance on the next trial. Note that each time we laid out cards on the table, we created a row with approximately equal distance between the cards to give children an intuitive understanding of how their estimate for the next trial related to their performance on the previous trial (in this way, we did not need to rely on their number sense). The experimenter told children “Now let's try again. We will use cards with different pictures on them this time.” The procedure was identical. Thus, children first indicated how many picture cards they thought they could remember this time, after which they studied the new set of picture cards for 15 s, and recalled as many pictures as possible. Again, the experimenter recorded the number of correctly recalled picture cards (i.e., Memory Self Performance 2). This procedure was repeated until participants had made four estimates and we had recorded three memory performances. Again, we needed an extra estimate after the last memory performance to be able to test whether children learn from their previous performance.

Immediately after participants completed the task, we showed them a video in which a peer of about the same age, sex, and nationality performed the same task. The experimenter paused the video just before the child in the video was about to recall the picture cards, and asked: “How many picture cards do you think […] can remember? As before, just leave the same number of blank cards to indicate how many pictures you think (s)he can remember.” The second experimenter recorded the number of blank cards left on the table (i.e., Memory Peer Estimate 1) and immediately removed all cards. Next, the experimenter assisted participants to watch the video of the peer's performance. They placed picture card(s) face down on the table to provide the participant with feedback on the peer's performance (i.e., Memory Peer Performance 1).

As in the motor task, the number of cards we laid out on the table to indicate the peer's performance matched the number of cards that participants themselves had correctly recalled in the corresponding trial (i.e., Memory Peer Performance 1 = Memory Self Performance 1; Memory Peer Performance *n* = Memory Self Performance *n*). With the face‐down picture card(s) still on the table, we asked participants again to leave blank cards on the table to indicate how many pictures they thought the child in the video would remember (i.e., Memory Peer Estimate 2). Then, the experimenter removed all the cards, assisted participants to watch the video of the peer's performance, and again placed a number of random picture card(s) face down on the table, matching participants’ own performance (i.e., Memory Peer Performance 2). We repeated this procedure until participants had made four estimates and had watched the peer in the video perform three trials.

## RESULTS

### Analytic strategy

We first conducted a series of descriptive analyses to test the equivalence of our samples and to explore potential sex and age effects for our main variables.

Next, to address our first hypothesis, we determined if children overestimated themselves on both tasks. We also explored potential cultural differences. We conducted a 2 (Performance Index: self‐estimated or actual) × 3 (Trial: 1, 2, or 3) × 2 (Nationality: Chinese or Dutch) repeated measures analysis of covariance (ANCOVA).

To address our second hypothesis, we determined if children would update their estimates of their own performance based on how they performed on prior trials, for both tasks. We conducted a 4 (Trial: 1, 2, 3, or 4) × 2 (Nationality: Chinese or Dutch) repeated measures ANCOVA, which allowed us to examine if children's performance estimates remained the same across trials (i.e., after receiving performance feedback). We also explored potential cultural differences. Furthermore, we explored correlations between children's actual performance on task trials and their subsequent performance estimates, as an additional test of whether they used performance feedback to inform their performance estimates.

To address our third hypothesis, we tested if children overestimated their own performance more than their peer's performance, on both tasks. For Trial 1, children's estimates of their own and their peer's performance cannot be meaningfully compared: Whereas children had no reference point to estimate their own Trial 1 performance, they did have such a reference point (i.e., their own performance) to estimate their peer's Trial 1 performance. Accordingly, to address this hypothesis, we compared performance estimates for Trials 2, 3, and 4. We first conducted a 2 (Performance Index: peer‐estimation or actual) × 3 (Trial: 1, 2, or 3) × 2 (Nationality: Chinese or Dutch) repeated measures ANCOVA to determine if children overestimated their peer's performance to begin with. Next, as a direct test of the third hypothesis, we conducted a 2 (Estimation Target: self or peer) × 3 (Trial: 2, 3, or 4) × 2 (Nationality: Chinese or Dutch) repeated measures ANCOVA to determine if children more strongly overestimated their own performance than their peer's performance. Again, we explored potential cultural differences.

The tests of the three hypotheses are confirmatory; they are based on previous empirical findings and have been pre‐registered. The tests of cultural differences (and the descriptive analyses) are exploratory; it is the first time that cultural differences in children's overestimation are examined.

### Descriptive analyses

Tables 1 and 2 present the descriptive statistics for children's performance estimates and actual performance on the motor and memory tasks.

**TABLE 1:** Children's self‐estimates, task performance, and peer‐estimates on the motor task

	All children		Chinese children		Dutch children	
	*M*	*SD*	*M*	*SD*	*M*	*SD*
Trial 1						
Self‐estimate	297.7	105.6	315.4	98.4	278.8	110.2
Task performance	116.4	45.7	106.9	38.1	126.5	51.0
Peer‐estimate	235.9	99.2	232.2	101.3	239.9	97.4
Trial 2						
Self‐estimate	218.8	103.4	210.0	102.4	228.2	104.3
Task performance	119.0	45.7	107.2	42.2	131.5	46.1
Peer‐estimate	224.1	102.4	220.0	102.5	228.4	102.5
Trial 3						
Self‐estimate	226.6	105.2	207.6	104.8	246.9	102.4
Task performance	129.3	50.8	117.7	44.9	141.6	53.9
Peer‐estimate	226.9	104.2	226.6	109.3	227.1	99.2
Trial 4						
Self‐estimate	233.1	108.3	218.3	106.4	248.7	108.6
Peer‐estimate	224.3	104.1	228.7	110.4	219.6	97.4

Note

Scores reflect distance in centimeters.

**TABLE 2:** Children's self‐estimates, task performance, and peer‐estimates on the memory task

	All children		Chinese children		Dutch children	
	*M*	*SD*	*M*	*SD*	*M*	*SD*
Trial 1						
Self‐estimate	5.55	4.17	6.17	4.72	4.87	3.36
Task performance	4.78	2.15	4.80	2.25	4.76	2.04
Peer‐estimate	7.66	4.36	8.40	4.63	6.85	3.91
Trial 2						
Self‐estimate	6.51	4.13	6.13	3.92	6.92	4.33
Task performance	3.67	1.80	3.91	1.79	3.41	1.78
Peer‐estimate	7.96	4.28	8.90	4.44	6.93	3.86
Trial 3						
Self‐estimate	7.09	4.20	6.80	4.31	7.42	4.07
Task performance	3.51	1.94	3.97	2.01	3.01	1.74
Peer‐estimate	8.12	4.26	9.17	4.22	6.96	4.02
Trial 4						
Self‐estimate	7.57	4.59	7.61	4.82	7.53	4.36
Peer‐estimate	8.06	4.51	9.16	4.39	6.85	4.35

Note

Scores reflect the number of cards remembered (possible range 0–15).

On average, Dutch children (*M* = 133.2) performed better than Chinese children (*M* = 110.6) on the motor task, *F*(1, 192) = 15.22, *p* < .001, $\eta_{p}^{2}$ = .073. Conversely, Chinese children (*M* = 4.23) performed better than Dutch children (*M* = 3.73) on the memory task, *F*(1, 189) = 4.92, *p* = .028, $\eta_{p}^{2}$ = .025.

Older children performed better on both the motor task (*r*s = .37–.43, *p*s < .001) and the memory task (*r*s = .24–.27, *p*s ≤ .001). Children's estimates of their own performance were mostly unrelated to age. As for children's estimates of their peer's performance, however, older children made more cautious estimates for most trials on the memory task (Trial 2–4: *r*s = −.15 to −.31, *p*s ≤ .033; Trial 1: *r* = −.06, *p* = .404), but not the motor task (*r*s = −.01 to .05, *p*s > .517). Because Chinese participants were slightly younger (i.e., 3 months) than Dutch participants, we included age as a covariate in all subsequent analyses.

On average, boys (*M* = 133.0) performed better than girls (*M* = 110.8) on the motor task, *F*(1, 192) = 14.62, *p* < .001, $\eta_{p}^{2}$ = .071. We found no sex difference for children's estimates of their own performance (*F*(1, 192) = 0.95, *p* = .330, $\eta_{p}^{2}$ = .005) or those of their peer's performance (*F*(1, 192) = 2.62, *p* = .107, $\eta_{p}^{2}$ = .013) on the motor task. As for the memory task, we found no sex difference in children's performance, *F*(1, 189) = 2.70, *p* = .102, $\eta_{p}^{2}$ = .014. However, boys did make more favorable estimates of their own performance (*M* = 6.99) than girls did (*M* = 5.79), *F*(1, 189) = 6.14, *p* = .014, $\eta_{p}^{2}$ = .031. Boys also made more favorable estimates of their peers’ performance (*M* = 8.56) than girls did (*M* = 7.27), *F*(1, 189) = 6.29, *p* = .013, $\eta_{p}^{2}$ = .032. Because of the sex differences we found, we also included sex as a covariate in all subsequent analyses.

### Do children overestimate their performance?

#### Confirmatory analysis

As hypothesized, children overestimated their performance on both tasks. For the motor task, there was a significant main effect of Performance Index, with children's estimates of their performance (*M* = 247.8) being more than twice as high as their actual performance (*M* = 121.8), *F*(1, 190) = 15.84, *p* < .001, $\eta_{p}^{2}$ = .077. This equals a self‐overestimation effect of 203%. For the memory task, there was a significant main effect of Performance Index as well, with children's estimates of their performance (*M* = 6.38) again being substantially higher than their actual performance (*M* = 3.97), *F*(1, 187) = 5.28, *p* = .023, $\eta_{p}^{2}$ = .027. This equals a self‐overestimation effect of 161%.

#### Exploratory analysis

**FIGURE 2:**
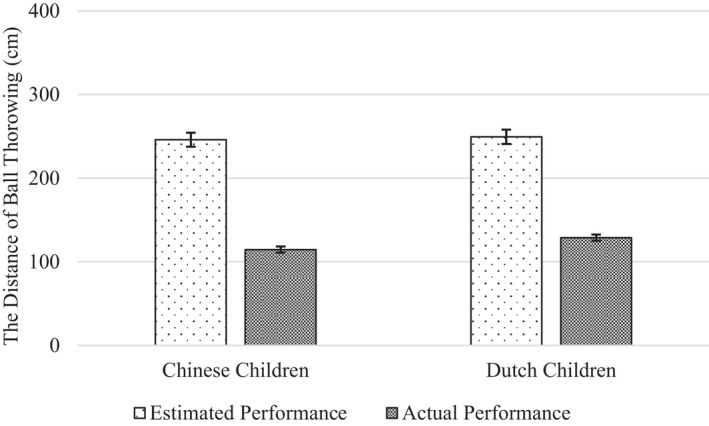
Chinese and Dutch children's estimated and actual performance on the motor task. *Note*: Error bars represent standard errors

**FIGURE 3:**
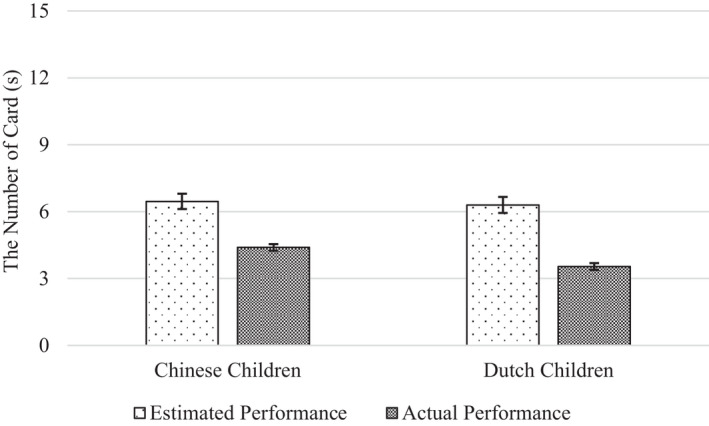
Chinese and Dutch children's estimated and actual performance on the memory task. *Note*: Error bars represent standard errors

We found no evidence for a cultural difference in the extent to which children overestimated their performance (Figures 2 and 3). The Performance Index × Nationality interaction was non‐significant, both on the motor task (*F*(1, 190) = 1.06, *p* = .305, $\eta_{p}^{2}$ = .006) and the memory task (*F*(1, 187) = 1.58, *p* = .211, $\eta_{p}^{2}$ = .008).

### Do children persist in overestimating their performance across trials?

#### Confirmatory analysis

As hypothesized, we found that children's estimates of their own performance, on both tasks, were relatively stable across trials. There were no significant main effects of Trial on the motor task (*F*(2.33, 442.35) = 2.81, *p* = .053, $\eta_{p}^{2}$ = .015), nor on the memory task (*F*(2.35, 439.20) = 2.62, *p* = .065, $\eta_{p}^{2}$ = .014). This finding suggests that, overall, children made little use of performance feedback to inform their subsequent performance estimates.

#### Exploratory analysis

**FIGURE 4:**
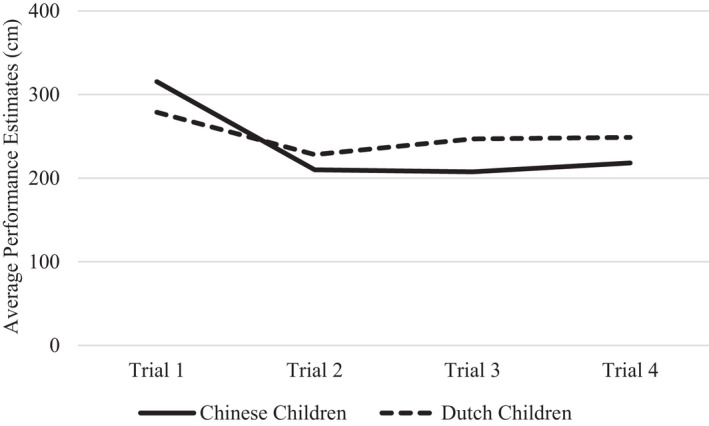
Chinese and Dutch children's performance estimates across trials on the motor task

**FIGURE 5:**
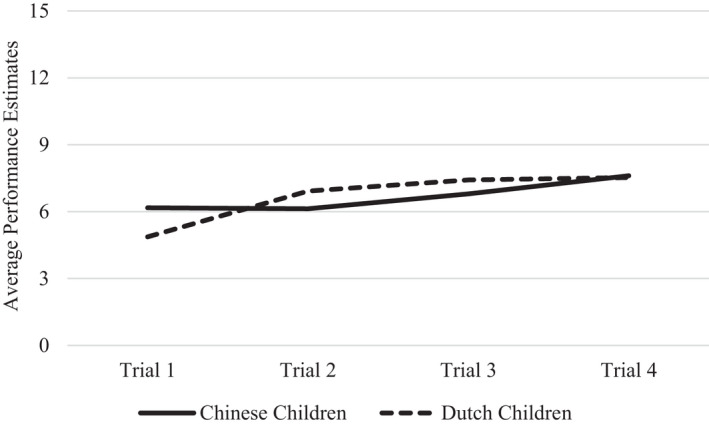
Chinese and Dutch children's performance estimates across trials on the memory task

We did find cultural differences. The Trial × Nationality interactions were significant for both the motor task (*F*(2.33, 442.35) = 7.85, *p* < .001, $\eta_{p}^{2}$ = .040), and the memory task (*F*(2.35, 439.20) = 4.32, *p* = .010, $\eta_{p}^{2}$ = .023). As Figures 4 and 5 show, cultural differences pertained mainly to the change that occurred from Trial 1 to 2. For the motor task, separate analyses for each nationality showed that Chinese children's performance estimates significantly decreased from Trial 1 to 2 (*F*(1, 97) = 4.89, *p* = .029, $\eta_{p}^{2}$ = .048). This decrease was smaller and not significant for Dutch children (*F*(1, 91) = 2.10, *p* = .150, $\eta_{p}^{2}$ = .023). For the memory task, Chinese children's performance estimates did not change from Trial 1 to 2 (*F*(1, 97) = 0.03, *p* = .856, $\eta_{p}^{2}$ = .000), whereas Dutch children's performance estimates even showed an increasing (rather than decreasing) trend, although this effect was not significant (*F*(1, 88) = 0.83, *p* = .365, $\eta_{p}^{2}$ = .009). After the second trial, Chinese and Dutch children's performance estimates remained largely stable, for both tasks.

To further explore the extent to which participants incorporated performance feedback into the estimates of their future performance, we inspected the pattern of correlations between children's task performance and their performance estimates on later trials (with age and sex partialled out), for both tasks.

As for the motor task (Table 3), the correlations between children's actual performance on a trial and their performance estimates for the subsequent trial were moderately positive and significant. This pattern of association is consistent with the possibility that children did, at least to some extent, make use of performance feedback to update their performance estimates on this task. Here, we found one difference between the Chinese and Dutch samples: The correlation between children's actual performance on the first trial and their performance estimate for the second trial was less strong in Chinese children as compared to Dutch children (Fischer's *Z* = −2.97, *p* < .01).

**TABLE 3:** Correlations between estimates and performance on the motor task

	Estimate2	Estimate3	Estimate4	Performance1	Performance2	Performance3
Estimate1	.41*** (.34**/.52***)	.25** (.24*/.33**)	.21** (.19/.28**)	.22** (.23*/.26*)	.17* (.12/.28**)	.16* (.11/.26*)
Estimate2		.61*** (.59***/.63***)	.63*** (.67***/.59***)	**.46** *** (**.26** ** **/.60** ***)	.49*** (.46***/.52***)	.35*** (.28**/.40***)
Estimate3			.73*** (.66***/.79***)	.38*** (.24*/.46***)	**.46** *** (**.47** *** **/.43** ***)	.46*** (.43***/.46***)
Estimate4				.30*** (.25*/.33**)	.43*** (.41***/.44***)	**.54** *** (**.53** *** **/.53** ***)
Performance1					.59*** (.41***/.72***)	.52*** (.42***/.57***)
Performance2						.66*** (.55***/.75***)

Note

Bold values are the correlations between performance on Trial *N* and estimate on Trial *N* + 1. Correlations for Chinese and Dutch children, respectively, are reported in brackets.

* *p* < .05

** *p* < .01

*** *p* < .001.

As for the memory task (Table 4), we found no such pattern of association. Here, children's actual performance on a trial and their performance estimates for the subsequent trial were not significantly correlated. In addition, for this task, we found no differences between the Chinese and Dutch samples.

**TABLE 4:** Correlations between estimates and performance on the memory task

	Estimate2	Estimate3	Estimate4	Performance1	Performance2	Performance3
Estimate1	.59*** (.71***/.52***)	.35*** (.49***/.14)	.32*** (.37***/.23*)	.02 (−.01/.05)	.09 (.02/.09)	.12 (.18/−.12)
Estimate2		.55*** (.77***/.32**)	.43*** (.63***/.22*)	**.02** (**−.07/.13**)	.01 (−.09/.12)	−.02 (.06/−.06)
Estimate3			.70*** (.79***/.59***)	−.15* (−.11/−.18)	**−.09** (**−.11/−.03**)	−.08 (−.11/.01)
Estimate4				−.15* (−.17/−.12)	−.12 (−.17/−.07)	**−.03** (**−.09/.06**)
Performance1					.41*** (.44***/.36**)	.37*** (.35***/.37***)
Performance2						.51*** (.42***/.54***)

Note

Bold values are the correlations between performance on Trial *N* and estimate on Trial *N* + 1. Correlations for Chinese and Dutch children, respectively, are reported in brackets.

* *p* < .05

** *p* < .01

*** *p* < .001.

### Do children overestimate their own performance more than their peer's performance?

#### Confirmatory analysis

Children overestimated their peer's performance on both tasks. On the motor task, a significant main effect of Performance Index indicated that children's estimates of their peer's performance (*M* = 229.0) were higher than their peer's actual performance (*M* = 121.8), *F*(1, 190) = 18.65, *p* < .001, $\eta_{p}^{2}$ = .089. This equals a peer‐overestimation effect of 188%. Similarly, on the memory task, a significant main effect of Performance Index indicated that children's estimates of their peer's performance (*M* = 7.87) were higher than their peer's actual performance (*M* = 3.97), *F*(1, 187) = 35.61, *p* < .001, $\eta_{p}^{2}$ = .160. This equals a peer‐overestimation effect of 198%.

**FIGURE 6:**
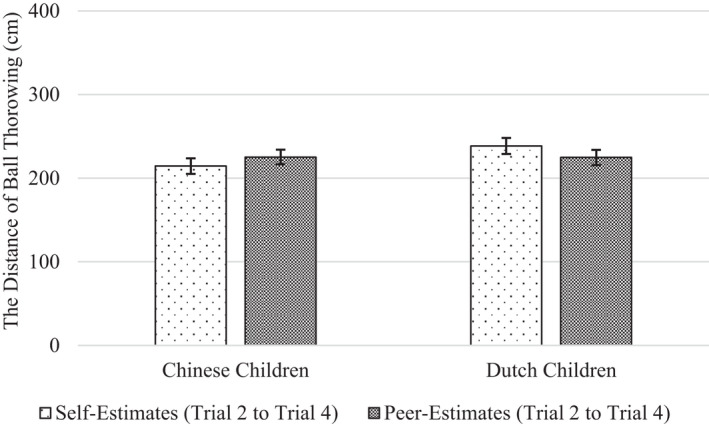
Chinese and Dutch children's self‐ and peer‐estimates on the motor task. *Note*: Error bars represent standard errors

**FIGURE 7:**
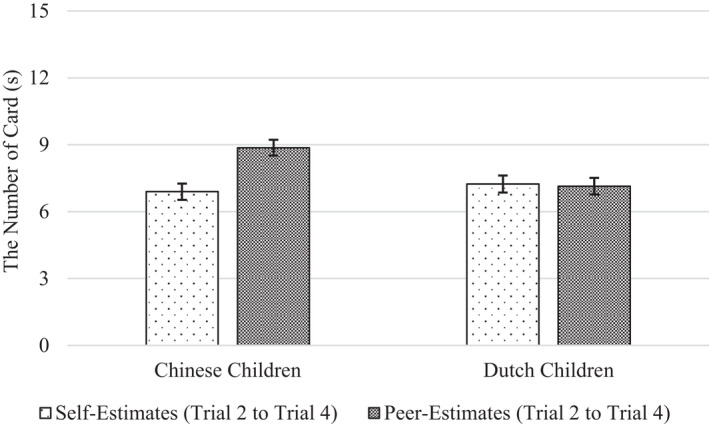
Chinese and Dutch children's self‐ and peer‐estimates on the memory task. *Note*: Error bars represent standard errors

Importantly, we found no support for the hypothesis that children would overestimate their own performance more than their peer's performance. On the motor task, there was no significant main effect of Estimation Target, *F*(1, 190) = 2.46, *p* = .119, $\eta_{p}^{2}$ = .013. Children's estimates of their own performance were about the same (*M* = 226.5) as their estimates of their peer's performance (*M* = 225.0). On the memory task, we did find a significant main effect of estimation target, but it was in the opposite direction of the hypothesis, *F*(1, 187) = 12.66, *p* < .001, $\eta_{p}^{2}$ = .063. Children estimated their own performance (*M* = 7.07) less (not more) favorably than they estimated their peer's performance (*M* = 8.01; Figures 6 and 7).

#### Exploratory analysis

We found cultural differences. On the motor task, Chinese and Dutch children differed in how they estimated their own performance relative to their peer's performance, *F*(1, 190) = 4.26, *p* = .04, $\eta_{p}^{2}$ = .022. Specifically, separate analyses for each nationality showed that whereas Chinese children estimated their own motor performance to be descriptively worse (*M* = 212.0) than the performance of their peer (*M* = 225.1), Dutch children estimated their own motor performance to be descriptively better (*M* = 241.3) than the performance of their peer (*M* = 225.0), although these differences in own versus peer performance estimates were not significant, *p*s > .08, $\eta_{p}^{2}$s < .033. We found a similar pattern for the memory task, *F*(1, 187) = 13.52, *p* < .001, $\eta_{p}^{2}$ = .067. Whereas Chinese children estimated their own memory performance to be significantly worse (*M* = 6.85) than that of their peer (*M* = 9.08), *F*(1, 97) = 10.32, *p* = .002, $\eta_{p}^{2}$ = .096, Dutch children estimated their own memory performance to be descriptively better (*M* = 7.29) than the performance of their peer (*M* = 6.91), *F*(1, 88) = 3.60, *p* = .061, $\eta_{p}^{2}$ = .039.

## DISCUSSION

We obtained evidence that Chinese 4‐ and 5‐year‐olds overestimate their performance on both a motor and a memory task about as much as their Dutch counterparts do. This finding suggests that young children's self‐overestimation is not a uniquely Western phenomenon. It can even be observed in a culture where children are socialized, from a young age, to refrain from self‐aggrandizement and show modesty (Q. Wang, 2004; Wu et al., 2002; Xu et al., 2005). Thus, our research suggests that the factors that push young Chinese children to hold inflated expectations of their performance are more powerful than those that pull them to adhere to the modesty imperative.

We also examined psychological processes that may account for young children's self‐overestimation. We found that, by and large, both Chinese and Dutch children persisted in overestimating their performance across trials, even if salient performance feedback indicated that they did not perform as well as they anticipated. Prior work has shown that children in the preschool and early school years are able to make quite accurate postdictions: They generally remember their performance on a task when asked directly afterward (Lipko et al., 2009; Schneider, 1998). Moreover, they realize that their past performance can predict their future performance on the same task (Lipko‐Speed, 2013). Our findings are thus consistent with a view that despite these abilities, children do not fully incorporate performance feedback into their performance predictions (Lipko et al., 2009; Lipko‐Speed, 2013; Schneider, 1998).

And yet, we found two important qualifiers to this general pattern. First, on the motor task, Chinese (but not Dutch) children did lower their performance estimates after the first trial, on which they typically performed worse than they had predicted. Second, also on the motor task, both Chinese and Dutch children's performance estimates were associated with their performance in previous trials. This latter finding suggests that even if children generally persisted in self‐overestimation, they did make use of their experience to make somewhat more informed estimates of their performance on subsequent trials—at least on the motor task. We conclude that it is not monitoring deficiency, but rather, incorporation inconsistency that contributes to young children's self‐overestimation: Children ages 4 and 5 fail to consistently or fully incorporate performance feedback into their performance estimates. This pattern was generally true for children from both nationalities, although Chinese children gave somewhat more evidence of realistically updating their performance estimates on the motor task than Dutch children did.

Our results are inconsistent with the wishful thinking account for young children's self‐overestimation. According to this account, preschoolers and kindergarteners often fail to distinguish performance wishes and expectations. One would expect, then, that their desire to be competent should positively bias their estimates of their own performance, but not those of an unknown peer's performance (because they have little investment in the peer's success; Stipek & Hoffman, 1980; Stipek et al., 1984). In the research design that we used, it was possible for such an effect to occur. Much like young children can use their peers’ performance as a reference point to make informed estimates of their own performance (Plumert & Schwebel, 1997), in this study, children could use their own performance as a reference point to realistically estimate their peer's performance. And yet, this is not what they did—instead, both Chinese and Dutch children generally overestimated their peer's performance about as much as their own. In fact, on the memory task, Chinese children overestimated their peer's performance even more (not less) than they overestimated their own.

Prior work did find that under some conditions, (Western) children make more accurate performance estimates when judging a peer than when judging themselves (Schneider, 1998; Stipek & Hoffman, 1980; Stipek et al., 1984). And yet, some of the same work showed that such discrepancies between self‐and peer‐estimates are conditional upon task characteristics, and such factors as the salience of past performance (Lipko et al., 2009; Schneider, 1998; Stipek et al., 1984). Together, this evidence corroborates the view that children exhibit a general positivity bias in their judgment of attributes and abilities—at least from the preschool age, they attend to, process, and interpret information selectively to maintain positive views of both themselves and others (Boseovski, 2010). We even found that Chinese (but not Dutch) children sometimes overestimate their peer's performance more than their own performance. This finding may be another manifestation of Chinese children's tactical self‐presentation—they possibly anticipated that making positive predictions about a peer would reflect well on them. Even then, our overall pattern of findings—including those in Dutch children—suggests that wishful thinking did not contribute to children's self‐overestimation.

An overarching question that emerges from these findings pertains to the consequences of young children's self‐overestimation. Research has demonstrated some potentially negative consequences: To the extent that children more strongly overestimate, specifically, their physical ability, they may be at increased risk of accidental injury (Plumert, 1995; Plumert & Schwebel, 1997). Nevertheless, there may also be important benefits to children's self‐overestimation that transcend cultural boundaries. Indeed, it has been argued that some aspects of cognitive immaturity, including self‐overestimation, have adaptive value (Bjorklund, 1997; Bjorklund & Green, 1992; Schwebel & Plumert, 1999). Given that young children have little experience with most activities they engage in, they could easily become discouraged or shy away from novel challenges if they accurately perceived the limits to their ability. Self‐overestimation may allow young children to feel efficacious despite their inexperience, to persist in the face of difficulty or failure, and to take on new challenges—thereby gaining important opportunities to develop abilities and improve performance (Shin et al., 2007).

### Strengths, limitations, and future research

Our research is the first to compare self‐estimates of performance in children growing up in a Western (i.e., the Netherlands) and non‐Western (i.e., mainland China) cultural context. The current literature on children's emerging self‐evaluation is heavily skewed toward samples of Western children, which raises questions about generalizability (Nielsen et al., 2017). This research provides a first step toward building a more culturally diverse understanding of children's self‐overestimation. We did so by building upon well‐established performance prediction methodological paradigms. To allow direct cross‐cultural comparison and avoid potential language confounds, we obtained non‐verbal performance estimates (i.e., placing flags, retaining cards), and also provided performance feedback using similar non‐verbal cues. We did so for both tasks, to maximize task comparability. Another methodological strength is that, in assessing peer performance estimates, we kept the alleged performance of the peer the same as the performance of the participant, to allow direct comparison unconfounded by differences in actual performance.

We also acknowledge limitations. We asked children to make self‐and peer‐estimates of performance in a fixed order (i.e., self‐estimates always preceded peer‐estimates). Indeed, our pilot study showed that it is difficult for children this age to estimate the performance of their peers with limited task experience, which is why we decided not to counterbalance. The implication, however, is that children's estimates of their peers’ performance may have been somewhat colored by their own experiences with the task.

Our findings suggest that young children do not consistently incorporate performance feedback into the estimates of their future performance. A valuable step for future research would be to provide an experimental test of this mechanism by comparing the performance estimates of children who do and do not receive feedback. Moreover, future research may test the developmental specificity of self‐overestimation by including older age groups in cross‐cultural comparisons. Research in Western samples suggests that self‐overestimation is pervasive in young children, but can sometimes be observed in older age groups as well. Future research will need to address the cultural generalizability of such observations.

In addition, research is needed to better understand both the malleability and adaptiveness of young children's self‐overestimation. For example, are there situational boundary conditions to self‐overestimation? To what extent is self‐overestimation rooted in socialization practices by parents? How do learning environments, and the extent to which they make salient individual achievement or normative evaluation (Dweck et al., 2014; Pang & Richey, 2007; Stipek & Daniels, 1988), influence children's self‐overestimation? And when or why is it adaptive for children to overestimate themselves? Insight in questions as these will be key to informing parenting experts and educators on how to help young children develop healthy views of themselves.

Finally, our findings should be interpreted in light of China's sociocultural change during the past few decades. Self‐enhancement is on the rise in China, a development that has been tied to socioeconomic transformation and changing cultural values (Cai et al., 2012; Zhang et al., 2017). Sociocultural change has been most pronounced in urban areas, where traditional cultural heritage now coexists with contemporary, individualistic values—a development which is echoed in evolving parenting practices, and has consequences for child adjustment (Chen & Li, 2012; Chen et al., 2009). We conducted our study in such an urban area—the city of Wenzhou. Thus, while the self‐overestimation of the Chinese children we studied was robust and substantial, future work will need to verify to what extent it can also be observed in children growing up in rural areas.

### Coda

Young children's self‐overestimation is not a uniquely Western phenomenon. Our research finds that non‐Western (i.e., Chinese) young children overestimate their task performance as much as their Western (i.e., Dutch) counterparts do. Moreover, children from both cultures persevere in overestimating themselves, despite receiving accurate performance feedback. Their rosy outlook on their own performance generalizes, though, to how they estimate the performance of their peers. In fact, Chinese children sometimes overestimate the performance of their peers even more than their own. Newborn calves are not afraid of tigers—indeed, they have high aspirations, both for themselves and for their peers.

## CONFLICT OF INTEREST

We have no conflicts of interests to disclose.

## Supporting information

Supplementary Material
