# Supplementary material for: Young children’s overestimation of performance: A cross‐cultural comparison
Source: Child Dev. 2021 Nov 6;93(2):e207–21. doi: 10.1111/cdev.13709 (PMC9298085; doi:10.1111/cdev.13709)
Supplement: Supplementary file 1 — Supplementary Material [file CDEV-93-e207-s001.zip › cdev13709-sup-0001-Preregistration (Anonymized).docx]

**Supplementary Material 1 – Original Preregistration**

**A Study on the Phenomenon of Children's Overestimation (#29787)**

Created: 10/24/2019 10:09 AM (PT)

1. **Have any data been collected for this study already?**

No, no data have been collected for this study yet.

**2) What's the main question being asked or hypothesis being tested in this study?**

This study investigates overestimation of performance in young children (i.e., 4 and 5 year old) in China and the Netherlands. We predict that: 1) Children’s estimates of their own performance are significantly higher than their actual performance, on both a motor task and a memory task. 2) Children overestimate the performance of a peer (of the same nationality and sex) significantly less than they overestimate their own performance. 3) Children’s overestimation of their own performance persists even after receiving accurate performance feedback (i.e., across trials) on both tasks; however, children's overestimation of peer performance does gradually decrease after receiving accurate performance feedback (i.e., across trials). We will additionally explore possible differences between Dutch and Chinese children for each of the above hypotheses.

**3) Describe the key dependent variable(s) specifying how they will be measured.** Participants’ estimation of their own performance, as well as their actual performance, will be measured in two tasks. In the motor task, participants are asked to throw a ball as far as they can. First, participants predict their performance by putting a green flag on the throwing field. The distance from the green flag to the starting line will be recorded as participants’ predicted performance. Next, participants throw the ball. The position where the ball first lands will be marked by a blue flag, and the distance from the blue flag to the starting line will be recorded as participants’ actual performance. In the memory task, participants are asked to recall as many picture cards as they can from a set of 15 picture cards that they are asked to memorize. First, participants predict how many picture cards they will be able to recall, by reserving a certain number of blank cards on the table. Next, participants try to recall the picture cards they were asked to memorize. The number of cards that participants accurately recall will be recorded as participants’ actual performance. For each task, we will make four measurements of estimated performance, and three measurements of actual performance. Participants’ estimation of a videotaped peer’s (of the same nationality and sex) performance on the same motor and memory task will be measured as well, using the same procedures. Here, for each task, we will make four measurements of estimated performance.

**4) How many and which conditions will participants be assigned to?**

The study is an observational study. All observations will be performed under the same conditions.

**5) Specify exactly which analyses you will conduct to examine the main question/hypothesis.**

1) Paired sample T tests will examine the hypothesized difference between children’s estimated and actual performance, for both tasks. 2) Paired sample T tests will examine the hypothesized difference between children's overestimation of their own and their peer’s performance, for both tasks. 3) Repeated measures ANOVAs will examine the hypothesized trends in overestimation across trials (i.e., a cross-trial decrease in overestimation of peers’ performance, but not own performance, for both tasks). We will additionally explore possible differences between Dutch and Chinese children for each of the above hypotheses, by including country as factor in the analyses.

**6) Describe exactly how outliers will be defined and handled, and your precise rule(s) for excluding observations.**

We will exclude data: •of participants who do not understand the task requirements, as judged by the experimenter; •of participants who do not follow the rules of the tasks, as judged by the experimenter; •of participants who fail to complete all three trials in the same task (although the data generated by the participant on other tasks will be retained if he/she did fully complete all trials in that task); •that deviate >3SD from the mean on the main study variables (i.e., the performance estimates and actual performances, for both tasks).

**7) How many observations will be collected or what will determine sample size?** **No need to justify decision, but be precise about exactly how the number will be determined.**

200 participants (i.e., 100 Dutch children, 100 Chinese children ). One of our aims is to explore whether Dutch and Chinese children make different predictions about task performance. We conducted a power analysis using G*power for this question, because it will require the largest sample size. To be able to detect an effect (i.e., difference between groups) of medium size (d=0.4), assuming power of 0.8, we will need a total sample of 200 participants (i.e., 100 Dutch children, 100 Chinese children).

**8) Anything else you would like to pre-register? (e.g., secondary analyses, variables collected for exploratory purposes, unusual analyses planned?)**

Nothing else to pre-register.
