# Supplementary material for: Young children’s overestimation of performance: A cross‐cultural comparison
Source: Child Dev. 2021 Nov 6;93(2):e207–21. doi: 10.1111/cdev.13709 (PMC9298085; doi:10.1111/cdev.13709)
Supplement: Supplementary file 1 — Supplementary Material [file CDEV-93-e207-s001.zip › cdev13709-sup-0002-Test cards.docx]

| No. | Theme | Set 1 | Set 2 | Set 3 |
| --- | --- | --- | --- | --- |
| 1 | Pets | Cat | Dog | Bird |
| 2 | Toys | Doll | Blocks | Soccer ball |
| 3 | Marine life | Starfish | Dolphin | Crab |
| 4 | Stationery | Pencil | Crayons | Scissors |
| 5 | Fruits | Grape | Banana | Strawberry |
| 6 | Transportation | Bike | Bus | Train |
| 7 | Tableware | Cup | Plate | Spoon |
| 8 | Appliances | Clock | Cell Phone | Television |
| 9 | Musical instruments | Piano | Drum | Violin |
| 10 | Vegetables | Carrot | Corn | Pumpkin |
| 11 | Livestock | Pig | Cow | Horse |
| 12 | Wildlife | Giraffe | Penguin | Elephant |
| 13 | Food | Cake | Noodles | Cookie |
| 14 | Furniture | Table | Chair | Sofa |
| 15 | Plant life | Flower | Tree | Grass |

**Set 1**


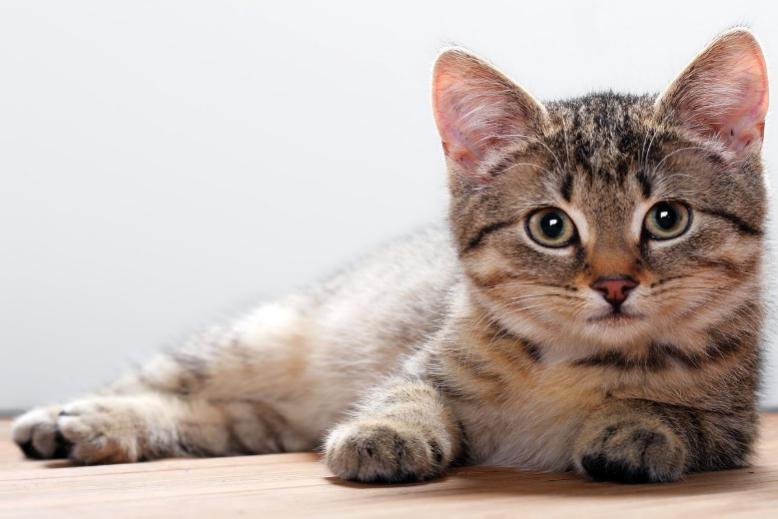

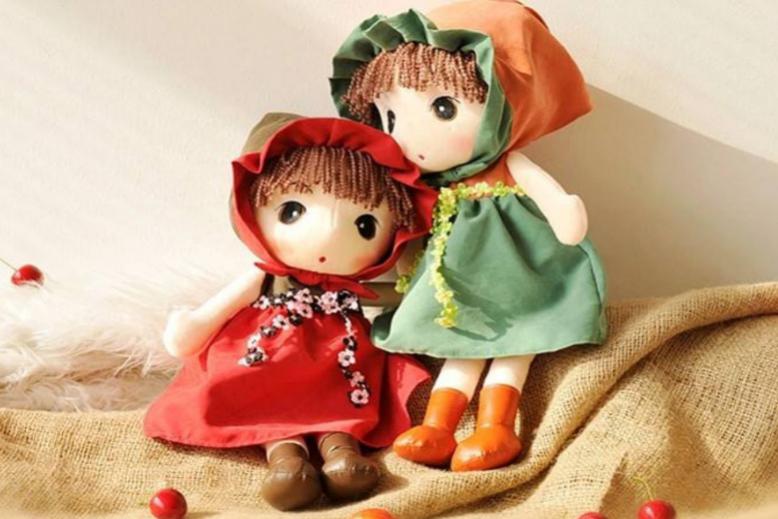

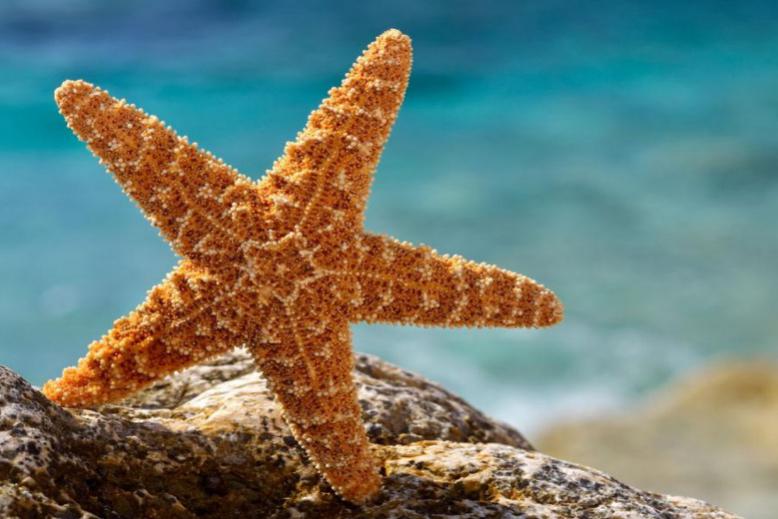
**
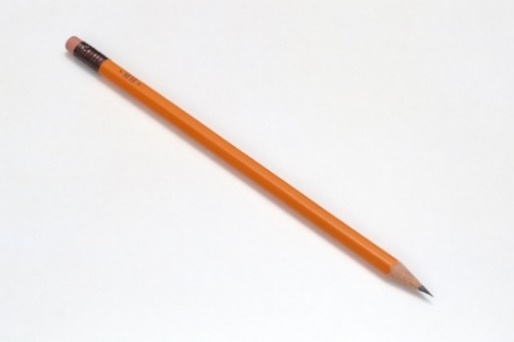
**
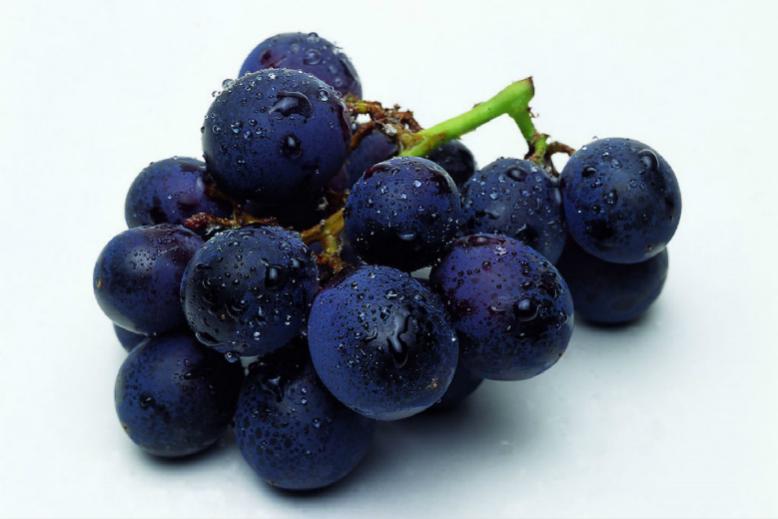
**
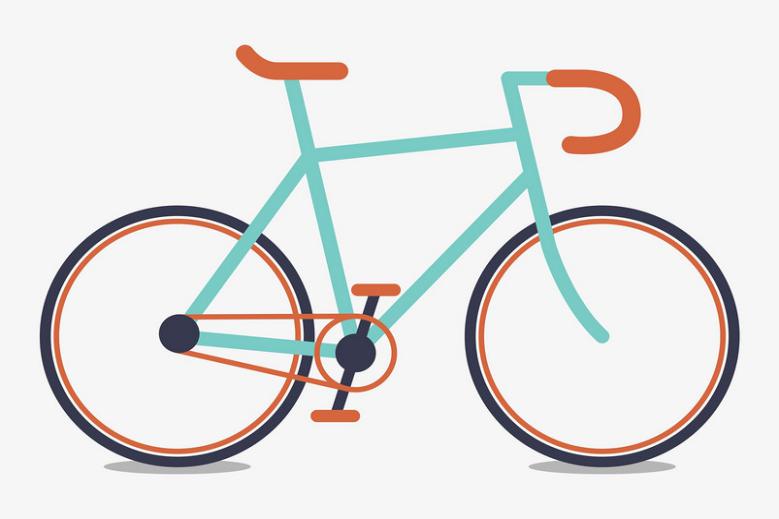

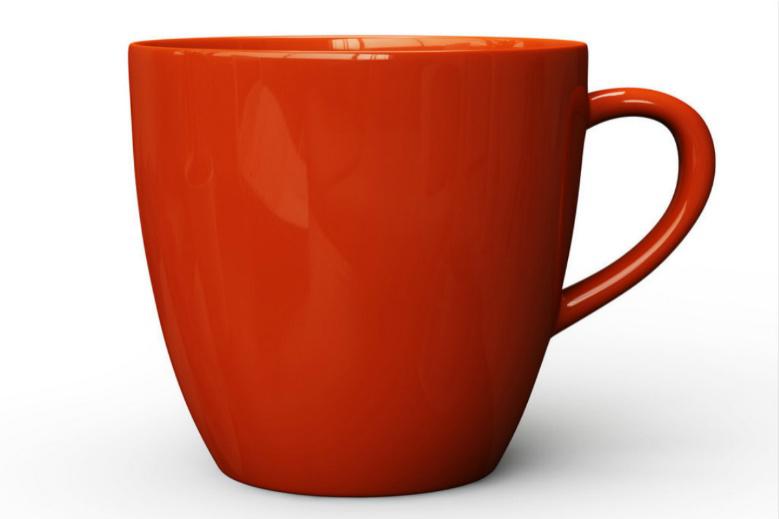

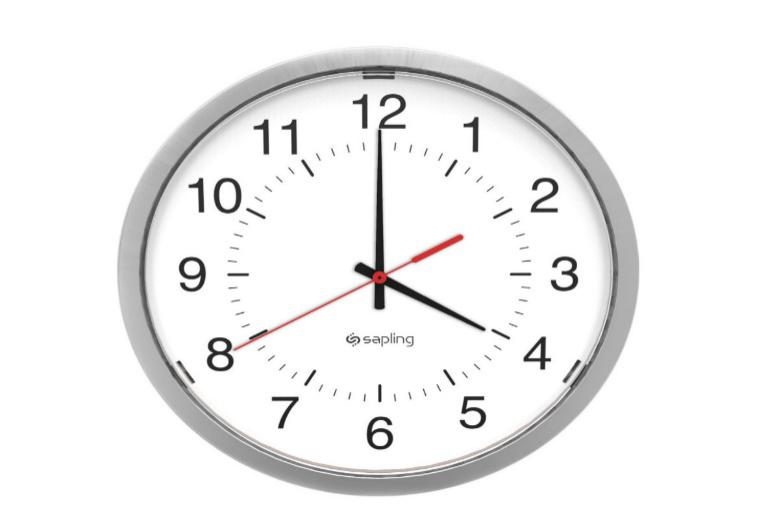

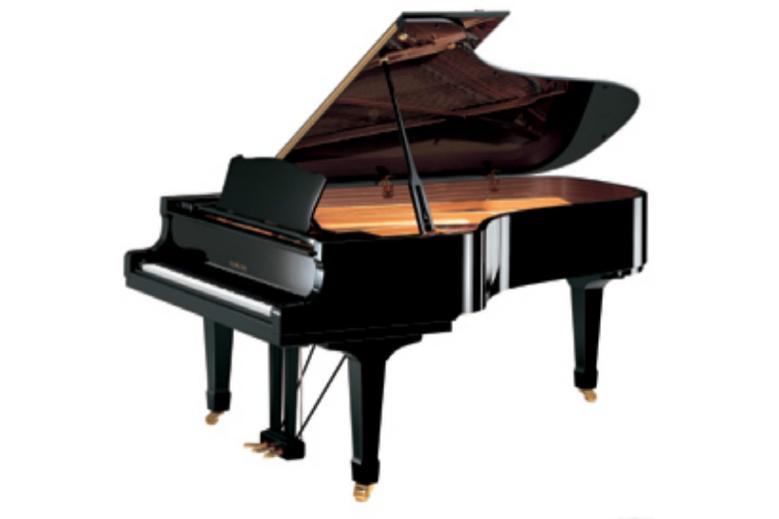

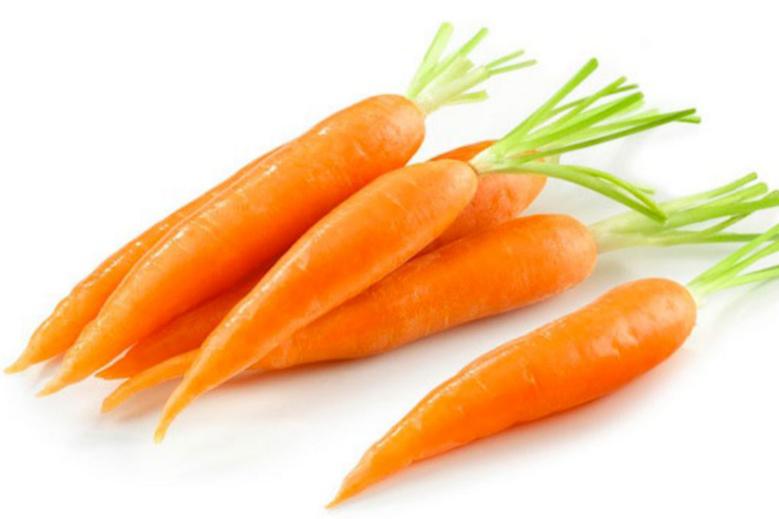

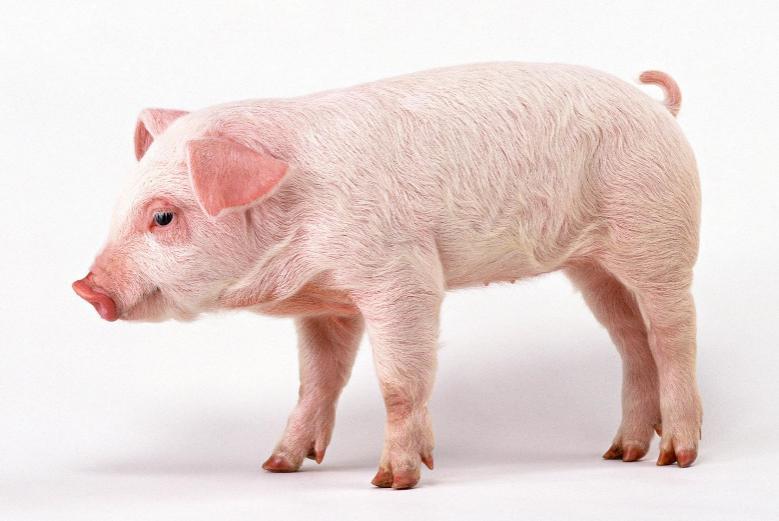

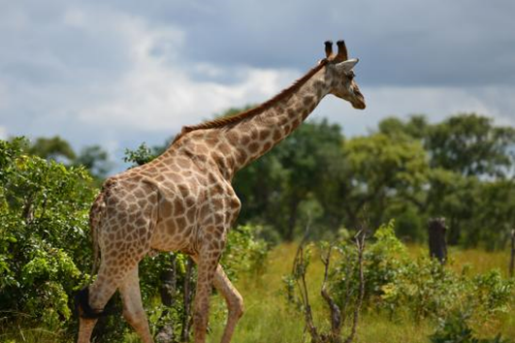

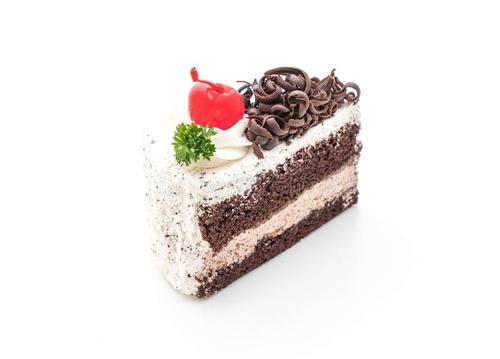

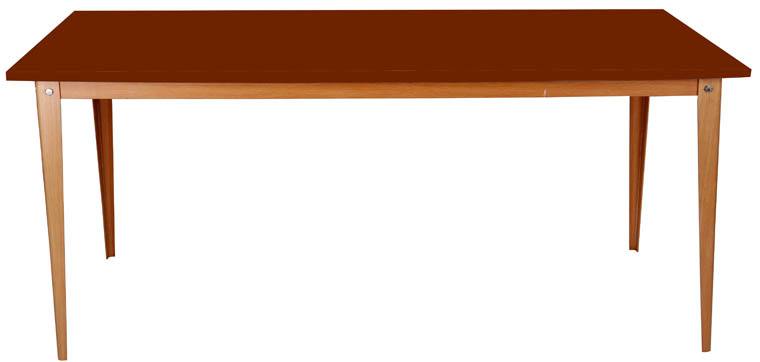

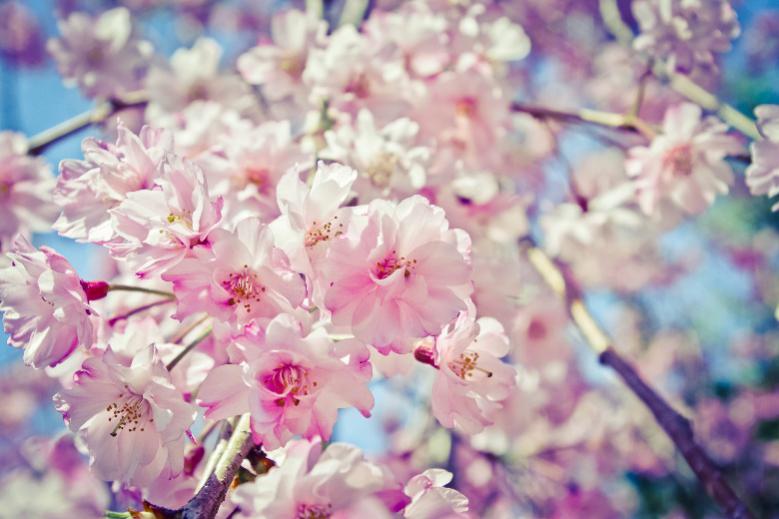
**

**Set 2**


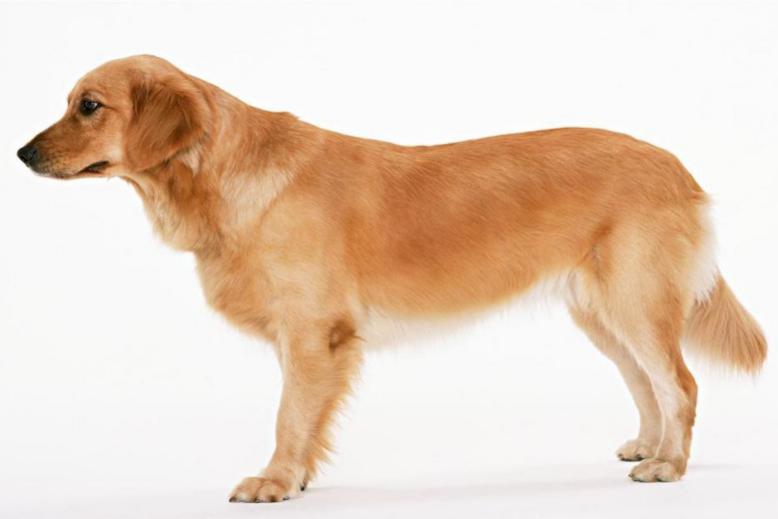
**
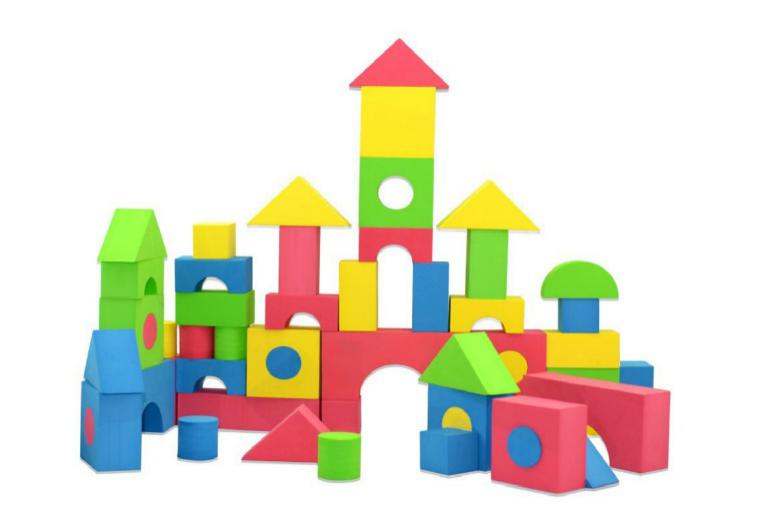
**
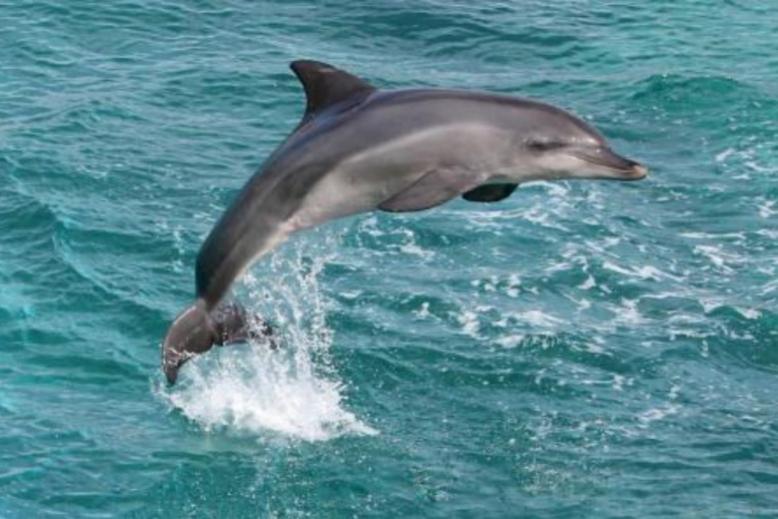
**
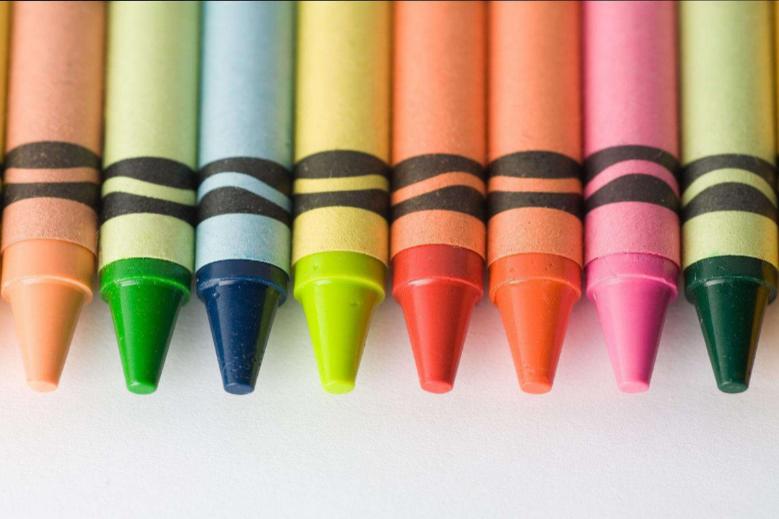
**
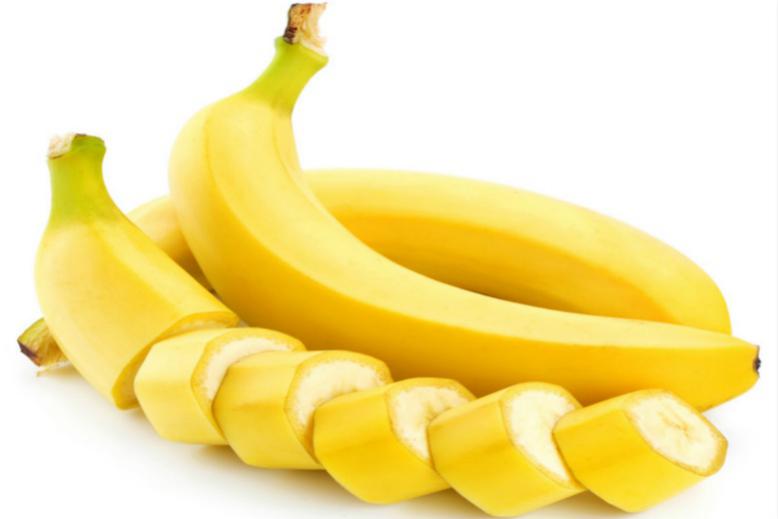
**
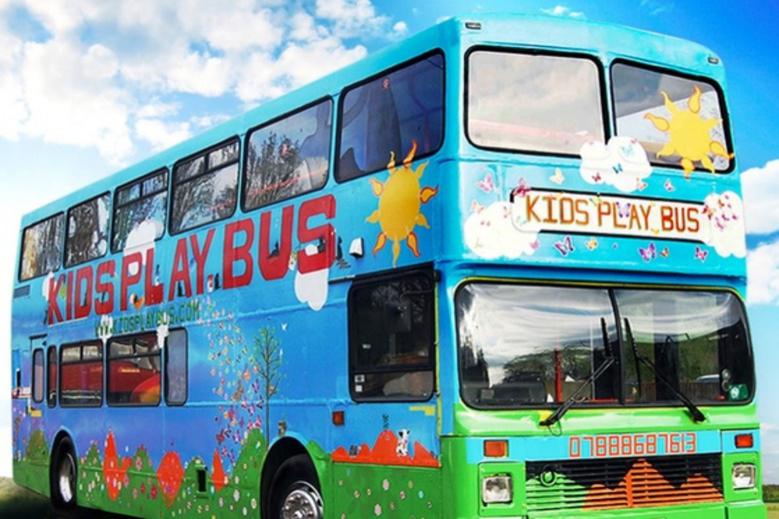

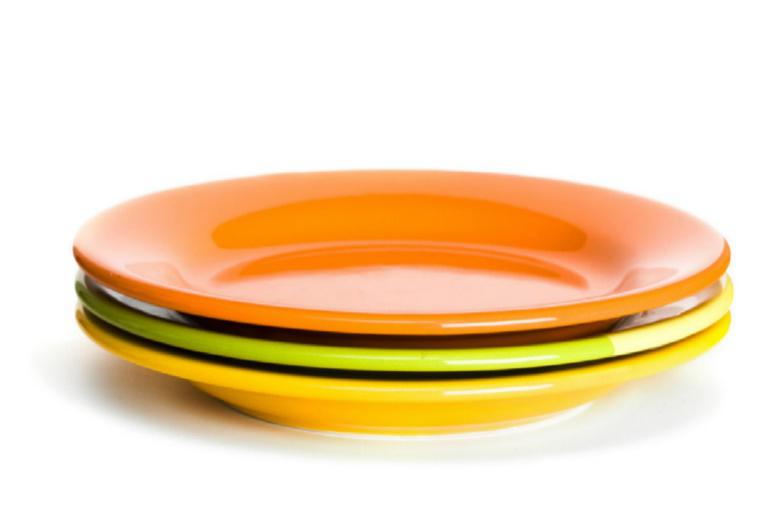
**
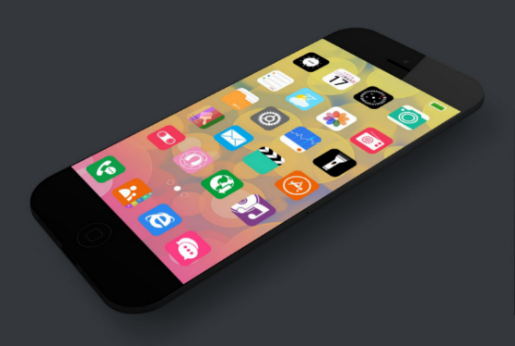

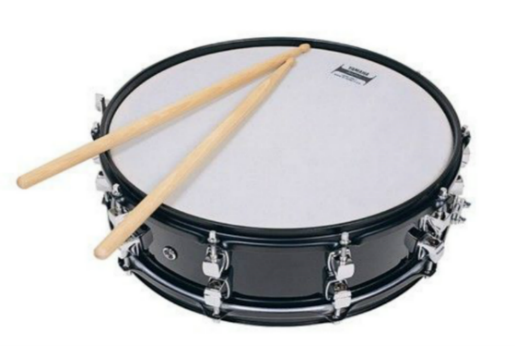
**
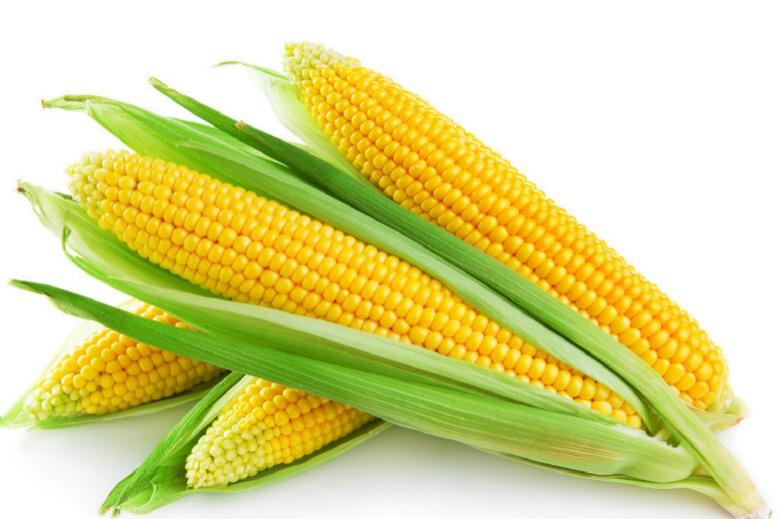

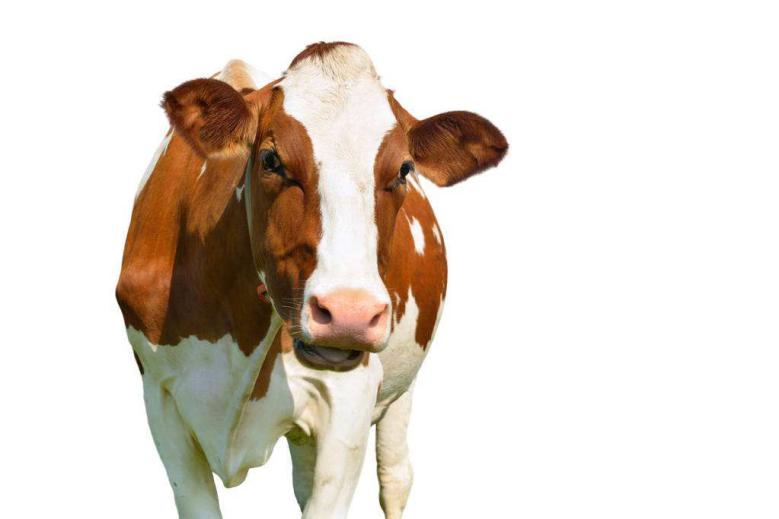
**
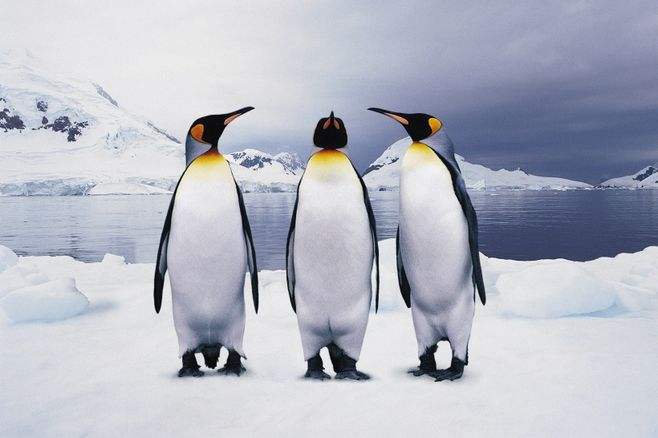
**
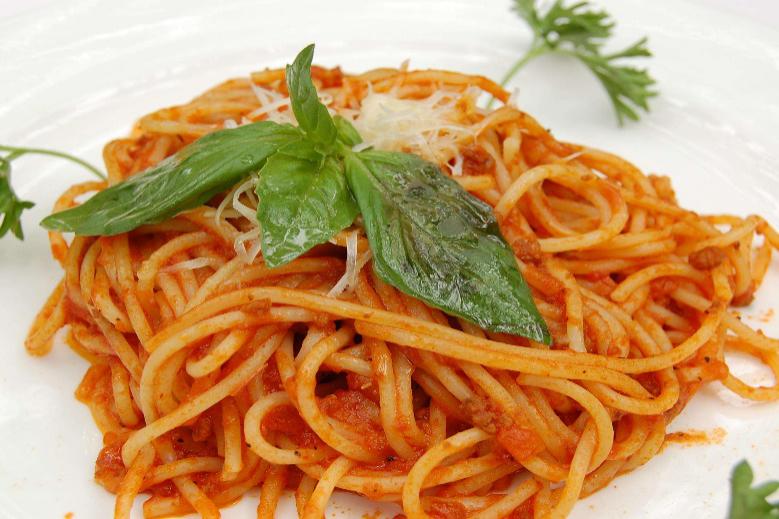

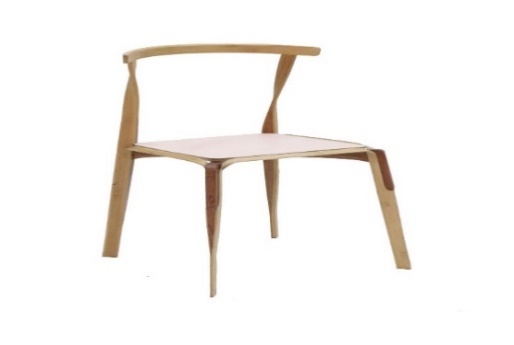

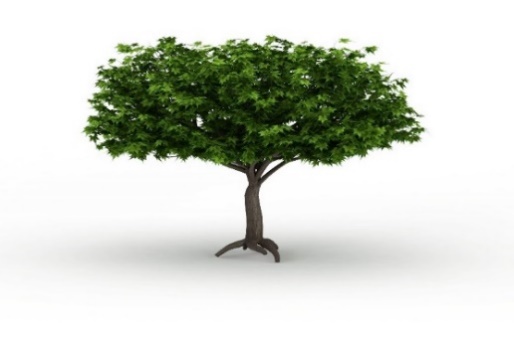
**

**Set 3**

**
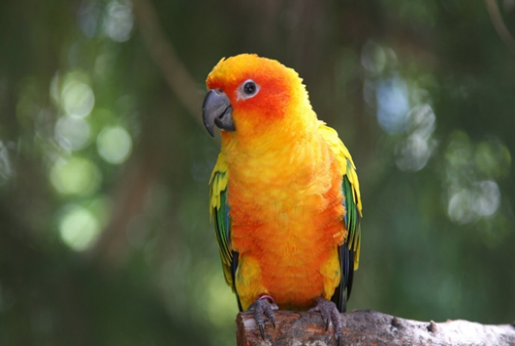
**
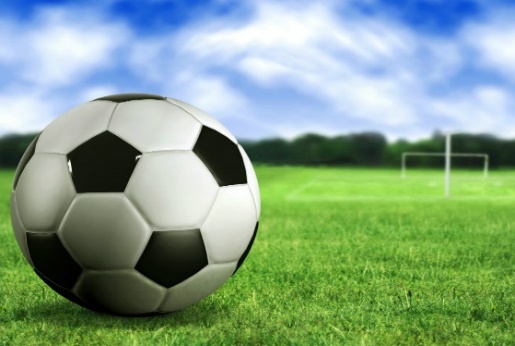

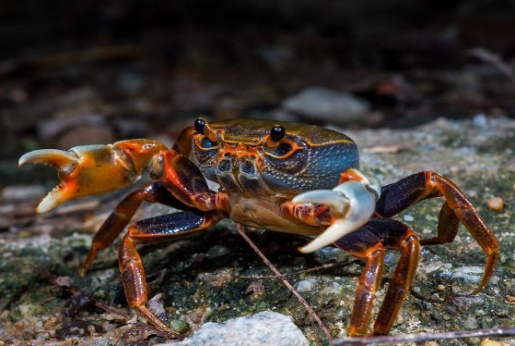
**
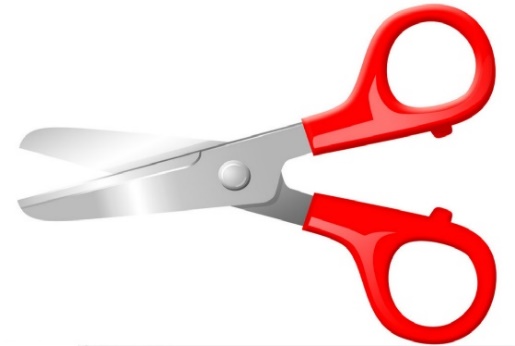

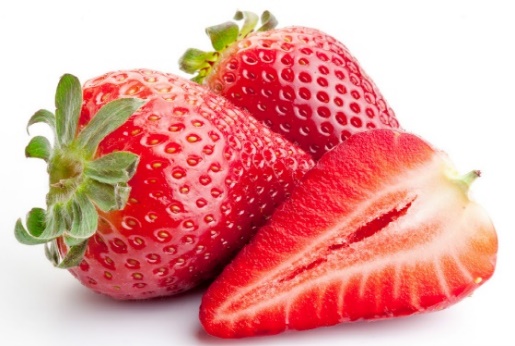

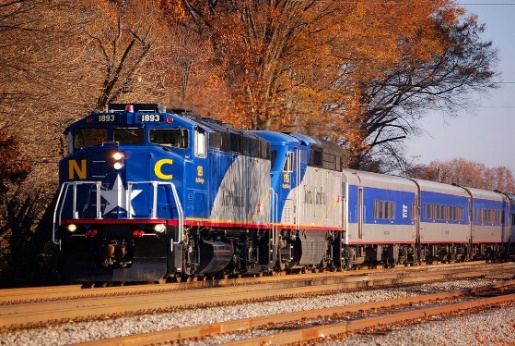
**

**
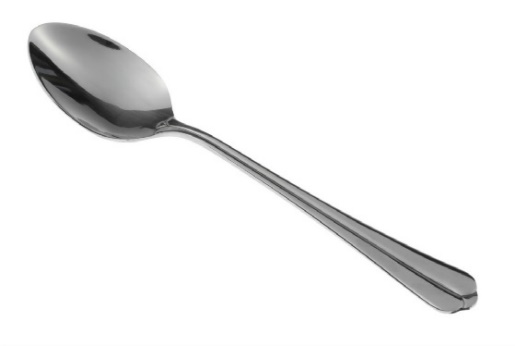

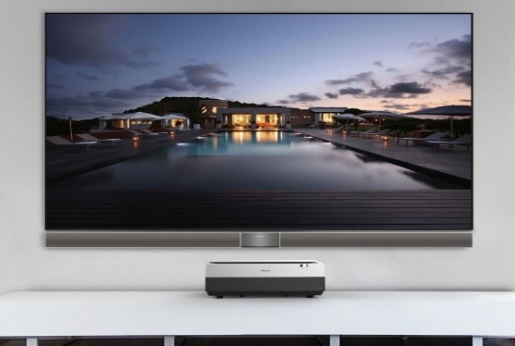
**
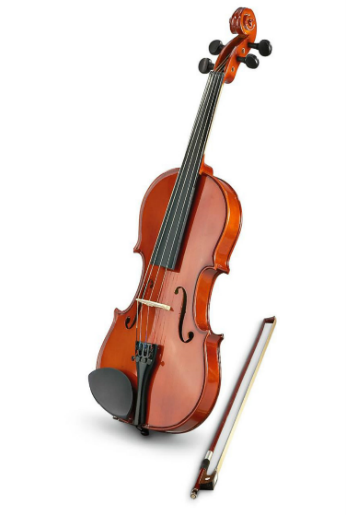


**
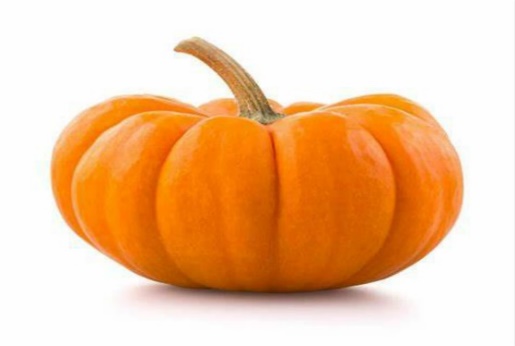

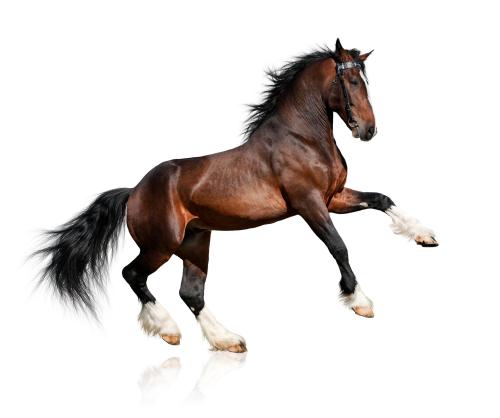

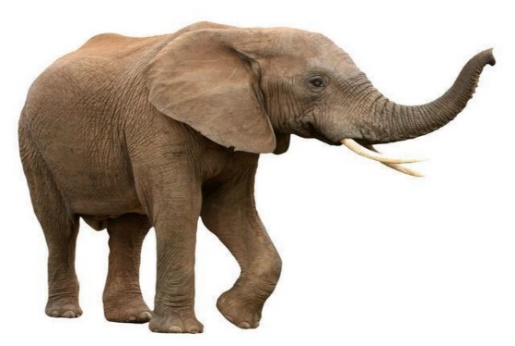

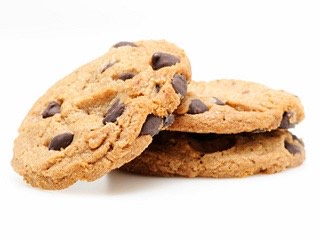

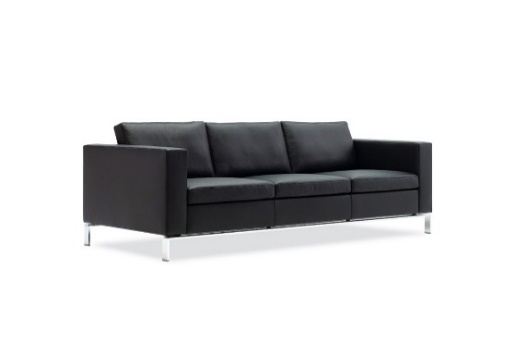

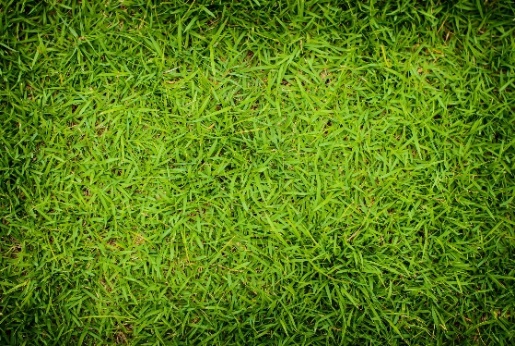
**
