# Supplementary material for: Young children’s overestimation of performance: A cross‐cultural comparison
Source: Child Dev. 2021 Nov 6;93(2):e207–21. doi: 10.1111/cdev.13709 (PMC9298085; doi:10.1111/cdev.13709)
Supplement: Supplementary file 1 — Supplementary Material [file CDEV-93-e207-s001.zip › cdev_13709_Deviations from preregistered analysis plan (AsPredicted #29787).docx]

**Supplementary Material 2 – Deviations From Preregistered Analysis Plan**

**Deviations From Preregistered Analysis Plan (AsPredicted #29787)**

The analyses reported in the article (‘Young Children’s Overestimation of Performance: A Uniquely Western Phenomenon?’) deviate from the analysis plan that we preregistered at aspredicted.org (#29787) in four ways. We list and explain the changes that we made here. We made change #1 before data analysis had begun. We made changes #2-4 after data analysis had begun.

Please note that what we labeled ‘hypothesis 2’ in the preregistration, is labeled ‘hypothesis 3’ in the article (and vice versa).

| **Change** | **Where?** | **What?** | **Why?** |
| --- | --- | --- | --- |
| 1 | Results | To test our preregistered hypotheses 1-3, we used repeated measures ANOVA analyses, rather than a series of T-Tests (preregistered hypotheses 1 and 2) or one-way ANOVAs (preregistered hypothesis 3). | We decided to use repeated measures ANOVA analyses to address our preregistered hypotheses 1-3 for two reasons. First, these analyses allow us to test the hypotheses both across samples (i.e., for Chinese and Dutch children together) and between samples (i.e., comparing Chinese and Dutch children). Second, these analyses provide a more conservative test of our hypotheses. We would have needed to test our hypotheses using separate T-Tests or one-way ANOVAs for each trial, thus increasing the risk of type 1 error. By using repeated measures ANOVAs, we kept the risk of Type 1 error low. |
| 2 | Results | We added a 2 (Performance Index: peer-estimation or actual) × 3 (Trial: 1, 2, or 3) × 2 (Nationality: Chinese or Dutch) repeated measures ANOVA before we tested our preregistered hypothesis 2. | In the preregistration, we assumed that children would overestimate the performance of their peers. We later decided that we would need to conduct an independent analysis to confirm this assumption. |
| 3 | Results | For our preregistered hypothesis 3, we did not analyze if children’s overestimation of their peers’ performance would gradually decrease across trials. | In the preregistration, we assumed that children would make more accurate estimations of their peers’ performance as compared to their own performance. We anticipated doing follow-up analyses to learn more about this discrepancy and test the possibility that it would be due to children gradually making more accurate estimations of their peers’ performance (but not their own performance) *across trials.* However, we found no support for the hypothesis that children would make more accurate estimations of their peers’ performance in the first place. This finding made further analysis superfluous. |
| 4 | Results | We added correlational analyses as an additional test of our preregistered hypothesis 3. Specifically, we inspected correlations between children’s actual performance on task trials and their performance estimations on subsequent trials. | We conducted these correlational analyses to provide further insight into whether children use performance feedback to inform their performance estimations. In doing so, we conform to an analytic approach that has been used before to address this question (Lipko et al., 2009). |
